# Influence of Alcohol Consumption on Body Mass Gain and Liver Antioxidant Defense in Adolescent Growing Male Rats

**DOI:** 10.3390/ijerph16132320

**Published:** 2019-06-30

**Authors:** Aleksandra Kołota, Dominika Głąbska, Michał Oczkowski, Joanna Gromadzka-Ostrowska

**Affiliations:** Department of Dietetics, Faculty of Human Nutrition and Consumer Sciences, Warsaw University of Life Sciences (SGGW-WULS), 159c Nowoursynowska Street, 02-776 Warsaw, Poland

**Keywords:** beer, ethanol, red wine, rats, body mass, liver, antioxidant defense, catalase, superoxide dismutase, glutathione

## Abstract

The World Health Organization (WHO) reported that alcohol consumption is a serious problem in adolescents. The aim of the study was to assess the influence of the time of exposure of various alcoholic beverages on body mass as well as on select parameters of liver antioxidant defense in adolescent Wistar rats. Thirty-day-old animals were divided into 12 groups (six animals in each): control and groups receiving various beverages containing 10% of alcohol (ethanol, red wine, beer), observed for two, four, and six weeks. The body weight gain and energy supply were analyzed for body mass assessment. The catalase (CAT), superoxide dismutase (SOD), glutathione peroxidase, transferase (GST), reductase activities, total antioxidant status, and glutathione level (GSH) were analyzed, for a liver antioxidant defense assessment. Group receiving red wine was characterized by the highest alcohol intake, lowest dietary intake, and highest total energy supply (*p* < 0.05). However, this did not influence body weight gain (*p* > 0.05). Reduced diet intake in groups receiving alcohol was counterbalanced by its energy value. Therefore, the energy supply was not lower than for the control (*p* > 0.05). Alcohol consumption and the experiment duration influenced CAT, SOD, and GST activities and GSH level. Alcohol consumption may influence hepatic antioxidant defense in adolescent male rats, but without influence on body weight gain.

## 1. Introduction

The Health Behavior in School-Aged Children (HBSC) survey [1]—a cross-national, collaborative study of the World Health Organization (WHO) conducted in 42 countries and regions—reported that alcohol use is a serious problem in adolescents. Every fifth adolescent over 14 years of age reports heavy episodic drinking, whereas 16% and 4% of boys aged 15 and 11 years old, respectively, declare they drink alcohol at least once a week [2]. The consequences of such frequency of alcohol consumption in adolescents include not only a risk of developing alcohol addiction [3], but also other health-related consequences, since the WHO indicated over 200 potential diseases [4]. Especially in young individuals, the results of regular alcohol consumption may be dangerous. The most important of these are the acute intoxication and chronic effects including liver cirrhosis, which has a steadily increasing frequency [5]. 

The majority of the ingested alcohol is metabolized in the liver, where highly reactive oxygen-containing molecules are generated [6], which causes oxidative stress [7]. Oxidative stress may directly damage cell structures, tissues, and organs, including the liver itself [8]. Moreover, oxidative stress may increase the generation of pro-inflammatory cytokines, such as the tumor necrosis factor α (TNF-α), which results in inflammatory processes [9]. With chronic alcohol consumption, both of these direct and indirect mechanisms are involved, which leads to the development of alcoholic liver disease [10]. 

A common consequence of alcoholic liver disease and both acute short-term and chronic alcohol overconsumption is malnutrition, which leads to decreased consumption of other food products, impaired digestion and absorption of nutrients, as well as increased metabolism and nutrient excretion [11]. Nutritional deficiencies lead to a decrease in body mass [12], despite the fact that, in general, alcohol may be a source of energy for the human body [13] and, therefore, its excessive consumption may lead to an increase in body mass [14]. Epidemiological studies have confirmed that, in adults, excessive alcohol consumption, especially between meals, often leads to the metabolic syndrome, with excessive body mass as one of its components [15].

Some mechanisms associated with alcohol use are well known in adults, even though the response of a developing organism to chronic excessive alcohol consumption is not known. Moreover, there were already a number of animal model studies conducted to analyze the influence of alcohol overconsumption on the hepatic damage during adolescence [16], or on the hepatic damage in progeny after exposure in mothers [17]. However, the studies in the group of adolescent organisms were conducted mainly in a model of binge drinking, as it was justified that binge drinking is the most common model of ethanol intake by adolescents [16] and only a few studies assessed prolonged drinking, but not for the liver parameters [18]. 

Taking it into account, the presented study was conducted with an aim to assess the influence of time of exposure of various alcoholic beverages on body mass as well as on select parameters of the liver antioxidant defense in adolescent Wistar rats.

## 2. Materials and Methods 

### 2.1. Animals and Experimental Procedures

The Local Ethics Committee for Animal Experiments in Warsaw, Poland (26/2007) approved all experimental procedures involving animals.

We obtained 72 pre-adolescent male Wistar rats (strain: Wistar Cmd: WI(WU)) initial body weight 93.5 ± 1.29 g) from the Mossakowski Medical Research Center of the Polish Academy of Sciences (Warsaw, Poland). There were three control and nine experimental groups that were assigned to various alcoholic beverages —three for ethanol, three for red wine, and three for beer. Before the main experiment, the weaned 21-day-old rats were housed in group cages (six animals per cage) and allowed to adapt to conditions at the vivarium for 10 days. During this period, animals from the experimental groups received solutions of alcoholic beverages (2%, 4%, 6%, 8%, and 10% of ethanol solutions, increased every second day) every day and had free access to water, as described in a previously published study [19]. At the end of the adaptation period, the 30-day-old rats underwent the experimental procedure. During the experiment, animals were housed individually in polypropylene cages under stable, controlled environmental conditions of 12:12 h of light:dark cycle, 50–60% humidity, and 23 ± 2 °C.

All animals had free access to a standard laboratory diet (Table 1).

Three groups received a standard diet and 10% aqueous ethanol solution, prepared from absolute ethyl alcohol (Chempur, Piekary Śląskie, Poland), for two, four, and six weeks. Three groups received a standard diet and dry red wine (Sophia Sakar, Cabernet Sauvignon, Bulgaria, 11% alc. vol.), which was diluted in tap water to obtain the final 10% ethanol solution, for two, four, and six weeks. Three groups received a standard diet and beer (Faxe Extra Strong, Royal Unibrew, Denmark), containing 10% ethanol, for two, four, and six weeks. Diet and alcoholic beverages were provided *ad libitum*. Experimental groups received only alcohol and no water during the dark cycle. In addition, during the day, they received water *ad libitum*. Intake of alcoholic beverages and diet was measured every day, whereas the study subjects were weighed once a week.

### 2.2. Sample Collection and Preparation of Liver Homogenates

At the end of the experimental period for each group (after two, four, or six weeks, the animals fasted overnight and were sacrificed by intraperitoneal administration of thiopental (120 mg/kg body weight). The livers from all rats were excised immediately, washed with physiological saline solution (0.9%), portioned, blotted dry, and stored at −80 °C. The tissues were homogenized in nine volumes of ice-cold 50 mM phosphate buffer (pH 7.4) containing 1M ethylenediaminetetraacetic acid (EDTA, Sigma-Aldrich, St. Louis, MO, USA). The homogenates were then centrifuged at 9000 *g* for 20 min at 4 °C (PRO 200, PRO Scientific Inc., Oxford, CT, USA), and the supernatant was pipetted into 1.5 mL Eppendorf tubes and frozen at −80 °C in aliquots until analysis.

### 2.3. Analytical Methods and Measurements

The catalase (CAT) activity was measured in liver homogenates using the method of Johansson and Borg [21] with the modification of Wheeler et al. [22]. 

The superoxide dismutase (SOD), glutathione peroxidase (GPX), glutathione transferase (GST), and glutathione reductase (GR) activities were measured using a spectrophotometric method in liver homogenates using the following kits: SD125 (Ransod, Randox Laboratories Ltd., Crumlin, UK) for the SOD, RS504 (Ransel, Randox Laboratories Ltd., Crumlin, UK) for GPX, CS0410 Glutathione S-Transferase (GST) Assay Kit (Sigma-Aldrich, St. Louis, MO, USA) for GST, and GR2368 (Randox Laboratories Ltd., Crumlin, UK) for GR.

The total antioxidant status (TAS) and glutathione level (GSH) were measured using a spectrophotometric method in liver homogenates using the following kits: NX2332 (Randox Laboratories Ltd., Crumlin, UK) for TAS and 703 002 Glutathione Assay Kit (Cayman Chemical Company, Ann Arbor, MI, USA) for glutathione level.

The results of CAT, SOD, GPX, and GR activities were expressed as units/mg of protein, GST activity as µM/min per mg of protein, TAS level as mM per mg of protein, and glutathione level as µM per mg of protein. Protein level in the homogenates of the liver were determined using the method by Bradford [23] using 110 306 Bioquant^®^ Protein (Merck, Darmstadt, Germany) and V900933 bovine serum albumin (Sigma-Aldrich, St. Louis, MO, USA) as a standard.

### 2.4. Statistical Analysis

Shapiro-Wilk test was used to verify the distribution of the obtained data. Due to the fact that, for the assessed parameters, both parametric and nonparametric distributions were observed, the results were presented as mean ± standard deviation (SD) and median values with minimum and maximum, in tables. For a parametric distribution, the one-way analysis of variance (ANOVA) was conducted with the Duncan’s post hoc test. For a non-parametric distribution, Kruskal-Wallis one-way ANOVA was used to compare groups. Statistical significance was considered for *p* ≤ 0.05. Statistical analyses were performed using the Statistica software version 13.1 (StatSoft Inc., Tulsa, OK, USA).

## 3. Results

### 3.1. Assessment of the Influence of Alcohol Consumption on Body Mass

Weekly body weight change (% of initial body mass) in the groups of experimental rats stratified by a group and experiment duration is presented in Table 2. There was no significant between-group difference in the weekly body weight gain. However, weight gain was found to be dependent on the duration of the experiment. Moreover, the initial body mass did not differ between the groups (*p* > 0.05).

Daily diet intake (kcal/day) in the groups of experimental rats stratified by a group and experiment duration is presented in Table 3. The daily diet intake differed significantly and independently from the experimental duration. The lowest intake was observed for red wine, and the highest intake was observed for the control group. At the same time, independently of the grouping, the lowest and highest daily dietary intakes were observed for two and four weeks of the experiment, respectively. 

Daily alcoholic beverage intake (kcal/day) in the groups of experimental rats stratified by a group and an experimental duration is presented in Table 4. The daily intake of the alcoholic beverage differed significantly and independently from the experimental duration. The highest and lowest intake were observed for the red wine and ethanol groups, respectively. Nevertheless, for the ethanol group, the longest experiment duration was associated with lower intake of alcoholic beverages.

The results of daily total energy supply (kcal/day) in the groups of experimental rats were stratified by a group and the experiment duration is presented in Table 5. The daily total energy supply differed significantly for the experimental duration for four and six weeks. For both durations, the highest supply was observed for red wine and ethanol, whereas the lowest was recorded for control and beer. Simultaneously, the maximum energy supply was observed after four weeks of the experiment. 

### 3.2. Assessment of the Influence of Alcohol Consumption on the Liver Antioxidant Defense

CAT activity (per mg of protein) in the groups of experimental rats was stratified by a group and the experiment duration is presented in Table 6. Compared to the groups stratified by the experimental duration, CAT activity differed significantly for the control group. The highest and lowest activities were observed after six and two weeks, respectively. For the experimental duration of four weeks, CAT activity differed between the groups, and the highest and lowest activities were reported for beer and the control group, respectively.

SOD activity (per mg of protein) in the groups of experimental rats was stratified by a group and the experiment duration is presented in Table 7. SOD activity differed significantly for the control and ethanol groups. The highest activity was observed after four weeks of the experiment, but the lowest activity was recorded for the control after two weeks and for ethanol after six weeks of the experiment. There were between-group differences for groups with an experiment duration of two and four weeks. After two and four weeks, the lowest activity was recorded for the control and beer group, respectively. 

GPX activity (per mg of protein) in the groups of experimental rats was stratified by a group and the experiment duration is presented in Table 8. No significant between-group differences were observed for GPX activity.

GST activity (µM/min per mg of protein) in the groups of experimental rats was stratified by a group and the experiment duration is presented in Table 9. GST activity differed significantly for the red wine group, and the lowest activity was observed after two weeks of the experiment. For the experiment duration of four weeks, GST activity differed between groups and the lowest and highest activities were reported for the beer and control groups, respectively.

GR activity (per mg of protein) in the groups of experimental rats was stratified by a group and experiment duration is presented in Table 10. No significant between-group differences were observed for GR activity.

TAS (mM per mg of protein) in the groups of experimental rats was stratified by a group and experiment duration is presented in Table 11. No significant between-group differences were observed for the TAS level.

The GSH level (µM per mg of protein) in the groups of experimental rats stratified by a group and experiment duration is presented in Table 12. GSH activity differed significantly for the control, ethanol, and red wine groups, and the highest activity was observed after four weeks of the experiment. For the experiment duration of four and six weeks, GSH activity differed between groups and the lowest and highest activity levels were recorded for beer and red wine, respectively. 

The summary of observed influence of the alcoholic beverage and experiment duration on the assessed parameters is presented in Table 13. All the previously described differences for CAT, SOD, and GST activity as well as the GSH level were included.

## 4. Discussion

### 4.1. Influence of Alcohol Consumption on Body Mass

In the present study, adolescent rats consumed more red wine, when compared to beer or ethanol. Despite a simultaneous lower dietary intake than the other groups, there was a significantly higher total energy supply for the red wine group than for the groups with other alcoholic beverages. However, this higher energy intake did not contribute to a higher body weight gain in comparison with the groups on other alcoholic beverages or the control group. The reduced dietary intake in the groups receiving alcoholic beverages was counterbalanced by the energy value of consumed alcohol. Moreover, the resultant total energy supply in the groups receiving alcoholic beverages was not lower than for the control groups. 

Though a decrease in weight gain was not observed for the groups receiving alcoholic beverages in our study, some studies have reported this to be caused by prolonged alcohol intake [24,25,26], including the murine studies [27]. Milat et al. [28] reported similar observations in a rat model, wherein one-month and three-month-old male rats received white wine (13% ethanol) for four weeks and a significantly lower body mass was recorded in comparison with their control group. According to the authors, non-phenolic compounds in white wine may have contributed significantly to the observed results via their influence on energy metabolism [28]. However, in this study, red wine, and not white wine, was used and, therefore, the observed differences may be a result of this fact.

Prolonged alcohol consumption may cause disturbances in the metabolism of macro-nutrients and micronutrients, including decreased digestion and absorption of impaired transport and utilization of nutrients, because of the influence of alcohol on the stomach [29] and intestines [30]. Simultaneously, alcohol consumption may increase the excretion of nutrients that may increase the risk of deficiency and malnutrition [24,31]. Therefore, prolonged alcohol consumption may result in lower body mass gain in animal experiments in comparison with animals that do not receive alcohol [32]. 

Regardless of whether acute or chronic intoxication effects are observed, both types of alcohol consumption may cause liver damage and impair amino acid uptake, protein synthesis, and protein transport from the liver [11]. Furthermore, these processes may influence the body mass.

In general, data on the influence of alcoholic beverages that are characterized by low ethanol content on the body mass are unambiguous and inconclusive. Negrão et al. [33] did not observe differences in body mass between adult male Wistar rats who were fed an ethanol solution or beer (both containing 5% ethanol) and those given no alcohol for five weeks. Similarly, Rodrigo et al. [34] reported the absence of between-group differences among adult male rats in a one-month study of ethanol solution or wine (both containing 12.5% ethanol) or those given no alcohol. However, in the study of Cowpland et al. [35], rats that received red wine containing 5% ethanol for nine weeks were characterized by lower body mass than those that received a solution of 5% ethanol or rats in the control group. This observation corresponds with an observed lower dietary intake in the present study that, nonetheless, did not contribute to a lower body mass due to the additional energy intake associated with the higher intake of an alcoholic beverage.

Furthermore, although young growing rats were studied in the present research work, adult animals are studied in a number of other studies, and that may have contributed to the observed differences between the results. Newbury-Birch et al. [5] indicated loss of appetite and a decrease in body mass among the consequences of ethanol consumption by young individuals. Although a decrease of body mass was not observed, and appetite was not studied in the present work, differences in dietary intake were observed and, for all the experimental groups, the intake was lower than that for the control group.

The processes of appetite and intake regulation are complex and, thus far, it was reported that ethanol may change the functioning of the nutrostat system by modulating the level of hormones participating in the regulation of dietary intake. Alcohol consumption may stimulate leptin [36] and decrease ghrelin production [37], whereas both these changes decrease appetite and may result in a body mass decrease. Simultaneously, in the case of growing organisms, the influence of alcohol consumption on growth hormone level may be especially important and may be decreased by ethanol consumption, with a resultant reduction in bodily growth [38].

Kokavec et al. [39] observed that various alcoholic beverages may have different influences on appetite. Red wine may increase appetite, whereas white wine may decrease it. However, at the same time, the potential influence of specific components, such as resveratrol in red wine [40] or xanthohumol in beer [41], on the risk of excessive body mass were observed. However, in the present study, whereas the intake of alcoholic beverage and total energy supply in a group receiving red wine was, in fact, higher than for the other groups, the dietary intake was lower. Thus, it may be inferred that a mechanism downregulating intake contributed to the lack of increase in body mass despite the additional energy supply from the intake of alcoholic beverages.

### 4.2. Influence of Alcohol Consumption on the Liver Antioxidant Defense

This study found that the consumption of alcoholic beverages as well as the experiment duration influenced CAT, SOD, and GST activity as well as the glutathione level in adolescent rats. However, the observed results differed for various alcoholic beverages and changed with the experiment duration, even though no stable trend was identified. Moreover, there were no between-group differences for GPX and GR activity as well as TAS.

The intake of alcoholic beverages has a complex influence on the body. Therefore, the prediction of metabolic changes, without any previous analysis, is not possible, and an explanation of the observed associations may be hindered. Tahir & Sultana [42] stated that the metabolism of alcohol causes hepatic oxidative stress that may be quantified on the basis of the decreased activity of both enzymatic (CAT, GPx, GR) and non-enzymatic antioxidative components (GSH). 

#### 4.2.1. Influence of Alcohol Consumption on CAT Activity

CAT is an enzyme that may be characterized by a peroxidative activity. Therefore, it may catalyze ethanol oxidation. Similarly, after four weeks of experiment in this study, animals consuming beer had increased CAT activity as compared with the control group. Such an influence may be attributed to the influence of some components of beer, which may be different than that of the ethanol itself, and these may have been lacking in red wine. Similarly, in a previous study by these authors, there was an evident lack of influence on the consumption of various wines on TAS [43]. In the present study, these findings may be inferred to possibly confirm the lack of influence of red wine consumption on some antioxidant defense parameters.

Xanthohumol, which is the major prenylchalcone in hops and beer, with demonstrated high antioxidant activity in inhibiting oxidation [44] may be responsible for such an influence, since it may take part in the reduction of oxidative stress, with a resulting increase in the activity of antioxidant enzymes. 

The specific influence of beer on CAT activity may be confirmed by the lack of such associations in the studies of other alcoholic beverages. In another rodent study where an ethanol solution containing 2, 4, or 6 g ethanol per kilogram of body mass was applied, no differences of CAT activity were observed as compared with the control group one hour after administration [45]. However, Scott et al. [45] hypothesized that the lack of observed differences may be attributed to lower alcohol intake because the metabolism of ethanol may not have generated excessive hydrogen peroxide, which is the main activator of the peroxidative activity of CAT. 

Furthermore, it may be supposed that the alcohol intake adopted in this study was too low, with the exception of beer where naringenin potentiated the influence. However, it may be inferred that the adaptive potential of the growing organism may have influenced the results we observed. This potential may be confirmed by the increased CAT activity in the control group (the highest, after six weeks of the experiment) that was attributed to intensified oxidative processes in the growing organism [46]. Moreover, such adaptive potential may have influenced the increase in CAT activity after four weeks of beer consumption, although not after six weeks.

#### 4.2.2. Influence of Alcohol Consumption on SOD Activity

The enzyme SOD participates in the antioxidative defense of the organism and cooperates with CAT. In this study, after four weeks of the experiment, increased CAT activity was reported simultaneously as decreased SOD activity in the group of animals consuming beer. However, a mere two weeks earlier (after two weeks of the experiment), the lowest SOD activity was recorded in a control group, and not in the group receiving beer. This higher SOD activity in all experimental groups, as compared to the control group, after two weeks of the experiment could have resulted from the disturbances of enzymatic mechanisms of antioxidative defense that may be reported even after a short exposure to alcohol. In addition, this was observed in the study of Scolaro et al. [47] in adult rats, after two weeks of ethanol solution intake, despite being at a higher concentration than in the present study (35% ethanol). The increase in SOD activity due to ethanol exposure may be associated with the prooxidative effect of ethanol, with a resultant increase in the production of reactive oxygen species as well as in the antioxidative defense of the organism [48]. In the presented study, for the group receiving ethanol for six weeks, the lower SOD activity was stated, which is consistent with the results of the other authors [49,50,51]. The observed situation may be due to an insufficient ability of the liver to remove free radicals, which leads to the oxidative stress. The other reason of decreased SOD activity may be inactivation of the enzyme by the free radicals being generated in the liver during the metabolic pathway of ethanol [45].

In beer, however, with the exception of ethanol, there are several compounds that may contribute to decreased SOD activity as compared to the other studied groups. Xanthohumol, which is one such example of these compounds in beer, decreases oxidative stress and may reduce the harmful hepatic influence of beer [52]. The study of Pinto et al. [52] revealed that an increase in xanthohumol intake for rats receiving ethanol was associated with a decrease in the SOD activity in the liver. Another component of beer that is characterized by antioxidative and anti-inflammatory properties is naringenin, which may influence antioxidative defense of cells by affecting not only SOD, but also other enzymes such as CAT, GPX, and GST [53].

Rodrigo et al. [34] did not find an influence of ethanol intake on SOD in adult Wistar rats that received a wine (12.5% ethanol). However, we emphasize that the present study evaluated growing, and not adult, animals. Moreover, differences during the experiment duration were observed that suggests the influence of the process of growth, whereas oxidative processes may have been elevated [46], which leads to a disturbance in the mechanisms of antioxidative defense that is also observed in the case of adolescents with metabolic disorders [54,55].

#### 4.2.3. Influence of Alcohol Consumption on the GSH Level

Another antioxidative compound, GSH, is the main non-protein thiol component. GSH plays a role in enzymatic reactions and, therefore, participates in the regulation of the function and structure of cells [56]. Gasbarrini et al. [57] did not observe any influence of beer or ethanol, administered for six weeks (dose 1.1 g/100 g of body mass), on the GSH level in the liver of rats. However, in this study, such differences were observed only after four and six weeks of the experiment, but not after two weeks. The lowest and highest GSH levels were recorded for beer and red wine, respectively. However, compared with the control group, the only differences were reported after four weeks of the experiment and only for the group receiving beer. There was a significantly lower GSH level than for the control group. As previously indicated, the differences observed for beer as compared with other alcoholic beverages may have resulted from the different composition and presence of specific bioactive components of beer. It was observed that some components, such as xanthohumol, may influence the GSH level [52]. Similarly, the specific results stated for red wine may be attributed to the influence of its specific components, including resveratrol that was observed to cause an increase in the GSH level [58]. However, they may be associated with the fact that a high dose of resveratrol may act not as an antioxidative compound, but as a pro-oxidant [59]. Therefore, for young rats, the applied dose may be crucial since it may induce a different effect than for older animals.

Another important issue is the applied dose of ethanol and the age of animals because, compared with the studies of other authors, various doses were applied and may have contributed to different conclusions. Roig et al. [60] indicated that administration of ethanol solution or sweet wine, both containing 20% ethanol, for six weeks caused a decrease of the GSH level in the liver of rats. In some other studies, lower GSH levels were observed in the liver than in the present study, but for the higher ethanol concentrations [49,50,61,62]. Authors surmise that the observed effect may be attributed to the binding acetaldehyde by GSH, with a resultant decrease in the GSH level. Furthermore, Das and Vasudevan [63] indicated that, in general, alcohol consumption induces a decrease of the GSH reserve and, thus, causes a reduction of antioxidative properties. However, DeLeve and Kaplowitz [64] concluded that a reduction in the GSH reserve of greater than 20% reduces the possibility of protecting cells against reactive oxygen species and causes damage to hepatocytes. 

#### 4.2.4. Influence of Alcohol Consumption on GST Activity

Among the enzymes that are dependent on GSH, GST participates in the removal of oxidized GSH from the cell after reduction of hydrogen peroxide. However, this enzyme removes xenobiotics from cells with the use of GSH in a coupling reaction [65]. In the present study, GST was influenced by the applied intervention and experiment duration. For the experiment duration of four weeks, GST activity differed between groups, and the lowest and highest activities were reported for beer and the control groups, respectively. This difference may be attributed, as previously indicated, to the influence of specific components of beer. However, due to a paucity of related studies, no potential influencing component could be indicated. Moreover, in groups stratified by the experiment duration, GST activity differed significantly for the red wine group, with the lowest activity observed after two weeks of the experiment. Such an observation may be the result of the defense mechanisms in the organism [60], but could also be attributed to a specific reaction of the developing organism to some bioactive components of red wine (e.g., resveratrol) that have, so far, been reported as being involved in detoxification [66]. Moreover, the general effect of wine consumption may be attributed to the overall mix of all its components and not to a specific ingredient [67]. However, this issue requires elucidation in future studies. 

The observed changes of the liver antioxidant defense were dependent on both the type of alcoholic beverage administrated and the experiment duration. Moreover, for other factors associated with the liver antioxidant defense, we did not observe any differences. Therefore, the influence of alcohol must be concluded to be multifaceted. Furthermore, it must be emphasized that only one dose of alcohol was assessed and the assessment was conducted for growing animals. Therefore, these results cannot be generalized. Despite the generally observed decrease in the activity of antioxidative enzymes after alcohol administration [11,68], it must be indicated that the observed effect depends on the age of animals, applied dose, experiment duration, and type of alcoholic beverage.

However, some limitations of the conducted study should be listed. It would be interesting to observe the energy expenditure of rats and whether their metabolism increased. The metabolic cages should be applied to observe it. For the standard cages in the conducted experiment, such observations were not possible. Taking this into account, further studies are needed.

## 5. Conclusions

The group receiving red wine was characterized by the highest alcohol intake, lowest dietary intake, and highest total energy supply. However, this did not contribute to higher gain in body weight when compared with other groups. The reduced dietary intake in the groups receiving alcoholic beverages was counterbalanced by the energy value of consumed alcohol. Therefore, the resultant total energy supply in the groups receiving alcoholic beverages was not lower than for a control group. The consumption of alcoholic beverages as well as the experiment duration influenced CAT, SOD, and GST activity, as well as the GSH level. However, no between-group differences were identified for GPX and GR activity or TAS. The consumption of alcoholic beverages may influence hepatic antioxidant defense in adolescent male rats, but there was no influence on body weight gain in the conducted experiment.

## Figures and Tables

**Table 1:** The composition of the applied standard laboratory diet.

Characteristics	Composition *
Energy value	Metabolisable energy—12.8 mJ; carbohydrates—60%; protein—30%; fat—10%
Basic composition	Crude protein—220.0 g; fat—42.0 g; crude fibre—35.0 g; starch—370.0 g; ash—9.5 g
Amino acids	Lysine—14.0 g; methionine—4.0 g; tryptophan—3.0 g; threonine—9.0 g; isoleucine—8.0 g; leucine—17.5 g; valine—10.0 g; histidine—4.0 g; arginine—13.0 g; phenylanaline—10.0 g; tyrosine—7.5 g
Minerals	Calcium—9.5 g; phosphorus—7.5 g; magnesium—2.9 g; potassium—9.5 g; sodium—2.0 g; sulfur—1.8 g; iron—150.0 mg; manganese—30.0 mg; copper—12.0 mg; iodine—0.3 mg; selenium—0.3 mg
Vitamins	Vitamin A—15,000 IU; vitamin D_3_—1000 IU; vitamin E—90.0 mg; vitamin K_3_—3.0 mg; vitamin B_1_—7.8 mg; vitamin B_2_—7.6 mg; vitamin B_6_—11.0 mg; vitamin B_12_—50.0 µg; pantothenic acid—22.0 mg; folic acid—2.0 mg; biotin—0.4 mg; nicotinic acid—60.0 mg; choline—1900 mg

* the composition based on the Nutrient Requirements of Laboratory Animals of the National Research Council [20] recalculated for 1 kg of the diet.

**Table 2:** Weekly body weight change (% of initial body mass) in the groups of experimental rats stratified by a group and experiment duration.

Group	Two Weeks		Four Weeks		Six Weeks	
	Mean ± SD	Median (Min–Max)	Mean ± SD	Median (Min–Max)	Mean ± SD	Median (Min–Max)
Control	155.08 ^c^ ± 6.65	153.84 (147.54–166.47)	195.96 ^b^ ±16.38	195.93 (168.61–219.79)	244.64 ^a^ ± 3.77	244.87 (238.48–248.58)
Ethanol	152.63 ^c^ ± 6.15	152.12 (143.82–161.59)	205.78 ^b^ ± 28.39	198.99 (173.52–253.65)	239.43 ^a^ ± 36.44	223.56 (206.29–305.48)
Red Wine	153.22 ^c^ ± 5.67	152.87 (146.62–160.26)	187.63 ^b^ ± 19.92	186.12 (155.59–216.29)	233.89 ^a^ ± 11.09	234.20 (219.71–248.18)
Beer	149.90 ^c^ ± 6.43	147.94 (144.44–161.16)	197.14 ^b^ ± 14.23	194.17 (180.57–222.67)	223.79 ^a^ ± 19.68	222.87 (199.15–245.93)

Different superscript letters indicate a significant difference between groups stratified by a group (A, B, C, D) and by experiment duration (a, b, c), for *p* ≤ 0.05.

**Table 3:** Daily diet intake (kcal/day) in the groups of experimental rats stratified by a group and experiment duration.

Group	Two Weeks		Four Weeks		Six |Weeks	
	Mean ± SD	Median (Min–Max)	Mean ± SD	Median (Min–Max)	Mean ± SD	Median (Min–Max)
Control	51.11 ± 4.77	52.58 *^Ab^ (50.51–63.43)	61.38 ^A^ ± 5.13	60.86 ^a^ (54.58–67.96)	60.24 ^A^ ± 1.49	60.28 ^ab^ (58.12–62.51)
Ethanol	49.91 ± 7.98	49.41 ^AB^ (41.11–60.97)	53.25 ^BC^ ± 2.20	52.42 (51.56–57.37)	48.64 ^B^ ± 3.47	49.19 (43.44–52.72)
Red Wine	46.06 ± 3.53	46.98 ^B^ (40.24–49.78)	49.47 ^C^ ± 3.87	50.06 (44.38–53.41)	49.29 ^B^ ± 3.33	48.87 (45.58–54.39)
Beer	47.55 ^b^ ± 8.19	50.28 ^AB^ (34.22–56.63)	56.01 ^Ba^ ± 5.21	55.55 (49.43–63.01)	53.03 ^Bb^ ± 5.53	54.31 (45.37–60.18)

*nonparametric distribution (assessed on the basis of the Shapiro-Wilk test, *p* ≤ 0.05. Different superscript letters indicate a significant difference between groups stratified by a group (A, B, C, D) and by experiment duration (a, b, c), for *p* ≤ 0.05.

**Table 4:** Daily alcoholic beverage intake (kcal/day) in the groups of experimental rats stratified by a group and experiment duration.

Group	Two Weeks		Four Weeks		Six Weeks	
	Mean ± SD	Median (Min–Max)	Mean ± SD	Median (Min–Max)	Mean ± SD	Median (Min–Max)
Ethanol	9.47 ^Ba^ ± 1.62	6.17 (4.95–7.68)	8.96 ^Ba^ ± 0.88	8.97 (7.66–10.28)	5.97 ^Cb^ ± 2.50	4.79 (3.89–10.20)
Red Wine	13.23 ^A^ ± 3.00	12.90 (9.94–18.41)	11.69 ^A^ ± 2.61	11.79 (8.64–15.04)	12.42 ^A^ ± 1.81	11.61 (10.73–15.64)
Beer	9.88 ^B^ ± 1.64	9.57 (7.67–12.36)	11.79 ^A^ ± 0.99	11.73 (10.79–13.59)	9.68 ^B^ ± 2.22	10.04 (5.61–11.78)

Different superscript letters indicate a significant difference between groups stratified by a group (A, B, C) and by experiment duration (a, b, c), for *p* ≤ 0.05.

**Table 5:** Daily total energy supply (kcal/day) in the groups of experimental rats stratified by a group and experiment duration.

Group	Two Weeks		Four Weeks		Six Weeks	
	Mean ± SD	Median (Min–Max)	Mean ± SD	Median (Min–Max)	Mean ± SD	Median (Min–Max)
Control	60.73 ^ab^ ± 9.41	58.81 (51.14–74.72)	62.22 ^Ba^ ± 2.51	61.43 (60.08–66.82)	54.61 ^Bb^ ± 4.71	55.62 (47.88–59.27)
Ethanol	59.29 ^b^ ± 4.72	59.89 (51.38–65.35)	72.84 ^Aa^ ± 7.55	69.81 (66.07–83.50)	61.71 ^Ab^ ± 3.75	61.48 (56.30–66.07)
Red Wine	57.43 ^b^ ± 7.44	59.68 (46.59–66.34)	67.79 ^ABa^ ± 4.47	66.79 (63.03–74.76)	62.71 ^Aab^ ± 7.28	64.14 (53.28–71.96)
Beer	60.73 ^ab^ ± 9.41	58.81 (51.14–74.72)	62.22 ^Ba^ ± 2.51	61.43 (60.08–66.82)	54.61 ^Bb^ ± 4.71	55.62 (47.88–59.27)

Different superscript letters indicate a significant difference between groups stratified by a group (A, B, C, D) and by experiment duration (a, b, c), for *p* ≤ 0.05.

**Table 6:** Catalase (CAT) activity (per mg of protein) in the groups of experimental rats stratified by a group and experiment duration.

Group	Two Weeks		Four Weeks		Six Weeks	
	Mean ± SD	Median (Min–Max)	Mean ± SD	Median (Min–Max)	Mean ± SD	Median (Min–Max)
Control	25.67 ± 1.12 ^b^	25.54 (24.31–27.67)	26.05 ± 5.78 ^Bb^	27.89 (15.66–31.72)	34.23 ± 5.52 ^a^	34.80 (25.91–42.85)
Ethanol	32.57 ± 8.34	33.91 (17.82–42.40)	33.85 ± 4.77 ^AB^	32.87 (28.20–40.41)	30.45 ± 10.74	30.11 (16.46–44.37)
Red Wine	34.58 ± 6.92	34.41 (22.85–43.01)	31.54 ± 6.26 ^AB^	31.23 (23.44–42.36)	35.27 ± 5.12	34.23 (29.44–43.55)
Beer	31.29 ± 10.54	32.72 (12.27–43.53)	36.75 ± 8.27 ^A^	35.68 (25.38–49.99)	35.76 ± 7.83	38.16 (23.72–44.39)

Different superscript letters indicate a significant difference between groups stratified by a group (A, B, C, D) and by experiment duration (a, b, c), for *p* ≤ 0.05.

**Table 7:** Superoxide dismutase (SOD) activity (per mg of protein) in the groups of experimental rats stratified by a group and experiment duration.

Group	Two Weeks		Four Weeks		Six Weeks	
	Mean ± SD	Median (Min–Max)	Mean ± SD	Median (Min–Max)	Mean ± SD	Median (Min–Max)
Control	11.45 ± 4.85 ^Bb^	11.40 (5.67–18.07)	28.31 ± 12.68 ^ABa^	28.17 (14.43–43.09)	26.26 ± 12.06 ^a^	23.38 (15.10–42.27)
Ethanol	27.20 ± 14.51 ^ABab^	25.65 (12.91–50.43)	35.97 ± 17.98 ^Aa^	38.60 (14.09–61.44)	17.81 ± 4.18 ^b^	18.26 (11.06–23.31)
Red Wine	27.18 ± 12.86 ^A^	27.06 (9.77–47.81)	26.75 ± 6.38 ^AB^	28.65 (14.55–32.85)	26.44 ± 11.97	28.35 (9.76–43.22)
Beer	28.15 ± 14.89 ^A^	28.51 (11.49–43.35)	18.92 ± 7.76 ^B^	19.53 (8.87–26.59)	26.76 ± 7.81	26.97 (13.46–37.68)

Different superscript letters indicate a significant difference between groups stratified by a group (A, B, C, D) and by experiment duration (a, b, c), for *p* ≤ 0.05.

**Table 8:** Glutathione peroxidase (GPX) activity (per mg of protein) in the groups of experimental rats stratified by a group and experiment duration.

Group	Two Weeks		Four Weeks		Six Weeks	
	Mean ± SD	Median (Min–Max)	Mean ± SD	Median (Min–Max)	Mean ± SD	Median (Min–Max)
Control	503.61 ± 133.11	445.95 (368.99–674.99)	528.87 ± 225.90	540.12 (204.94–834.24)	578.02 ± 183.37	637.29 (299.58–757.96)
Ethanol	439.07 ± 112.84	422.47 (322.02–606.50)	553.59 ± 130.48	548.59 (353.19–727.49)	592.77 ± 215.75	522.07 (366.48–863.96)
Red Wine	382.94 ± 164.53	364.19 (161.83–638.57)	568.04 ± 237.41	533.19 (322.63–856.56)	575.64 ± 147.01	560.21 (429.46–826.90)
Beer	533.60 ± 287.11	475.1 (230.85–1062.58)	457.91 ± 207.22	533.38 (125.90–677.31)	483.07 ± 164.78	493.12 (232.88–725.71)

Different superscript letters indicate a significant difference between groups stratified by a group (A, B, C, D) and by the experiment duration (a, b, c), for *p* ≤ 0.05.

**Table 9:** Glutathione transferase (GST) activity (µM/min per mg of protein) in the groups of experimental rats stratified by a group and experiment duration.

Group	Two Weeks		Four Weeks		Six Weeks	
	Mean ± SD	Median (Min–Max)	Mean ± SD	Median (Min–Max)	Mean ± SD	Median (Min–Max)
Control	2.69 ± 0.63	2.55 (2.13–3.68)	3.36 ± 0.83 ^A^	3.45 (2.22–4.61)	2.91 ± 0.39	2.88 (2.29–3.39)
Ethanol	2.97 ± 0.55	3.06 (2.09–3.67)	2.85 ± 0.39 ^AB^	2.81 (2.31–3.48)	3.08 ± 0.71	2.86 (2.39–3.95)
Red Wine	2.13 ± 0.38^b^	2.09 (1.67–2.62)	3.02 ± 0.48 ^ABa^	2.88 (2.33–3.64)	2.91 ± 0.52 ^a^	2.70 (2.45–3.69)
Beer	3.01 ± 1.18	2.83 (1.84–5.28)	2.43 ± 0.11 ^B^	2.43 (2.30–2.56)	2.67 ± 0.58	2.75 (1.97–3.54)

Different superscript letters indicate a significant difference between groups stratified by a group (A, B, C, D) and by the experiment duration (a, b, c), for *p* ≤ 0.05.

**Table 10:** Glutathione reductase (GR) activity (per mg of protein) in the groups of experimental rats stratified by a group and experiment duration.

Group	Two Weeks		Four Weeks		Six Weeks	
	Mean ± SD	Median (Min–Max)	Mean ± SD	Median (Min–Max)	Mean ± SD	Median (Min–Max)
Control	4.40 ± 1.22	4.16 *(3.05–6.68)	4.50 ± 0.47	4.59 * (3.90–5.01)	3.93 ± 0.78	3.67 * (3.13–5.32)
Ethanol	4.73 ± 0.95	4.64 *(3.52–6.39)	4.88 ± 0.70	4.68 * (4.31–6.18)	4.60 ± 1.37	5.13 * (2.10–5.75)
Red Wine	4.40 ± 1.22	4.16 *(3.05–6.68)	5.02 ± 1.18	4.67 * (4.07–7.15)	4.62 ± 0.97	4.38 * (3.82–6.40)
Beer	3.94 ± 0.68	3.99 * (2.75–4.79)	4.56 ± 1.09	4.93 * (2.49–5.48)	3.55 ± 0.96	4.09 (1.95–4.22)

* nonparametric distribution (assessed on the basis of the Shapiro–Wilk test, *p* ≤ 0.05). Different superscript letters indicate a significant difference between groups stratified by a group (A, B, C, D) and by experiment duration (a, b, c), for *p* ≤ 0.05.

**Table 11:** Total antioxidant status (TAS) (mM per mg of protein) in the groups of experimental rats stratified by a group and experiment duration.

Group	Two Weeks		Four Weeks		Six Weeks	
	Mean ± SD	Median (Min–Max)	Mean ± SD	Median (Min–Max)	Mean ± SD	Median (Min–Max)
Control	1644.86 ± 293.65	1611.95 * (1261.23–2145.27)	1827.04 ± 492.06	1783.10 * (1282.38–2624.11)	1510.27 ± 286.98	1623.15 * (1063.16–1791.32)
Ethanol	1408.05 ± 373.05	1315.19 * (1052.02–2065.23)	1524.45 ± 282.03	1387.91 * (1295.19–1983.86)	1353.52 ± 263.17	1286.96 * (1034.26–1716.87)
Red Wine	1578.42 ± 302.78	1639.43 * (1138.56–1890.76)	1742.36 ± 403.99	1781.96 * (1280.31–2397.19)	1673.22 ± 283.75	1633.18 * (1374.71–2014.41)
Beer	1549.22 ± 295.95	1463.22 * (1284.07–1943.74)	1478.79 ± 162.97	1508.03 * (1180.11–1678.73)	1373.64 ± 244.02	1373.76 * (1047.22–1699.19)

* nonparametric distribution (assessed on the basis of Shapiro–Wilk test, *p* ≤ 0.05). Different superscript letters indicate a significant difference between groups stratified by a group (A, B, C, D) and by experiment duration (a, b, c), for *p* ≤ 0.05.

**Table 12:** Glutathione (GSH) level (μM per mg protein) in the groups of experimental rats stratified by a group and experiment duration.

Group	Two Weeks		Four Weeks		Six Weeks	
	Mean ± SD	Median (Min–Max)	Mean ± SD	Median (Min–Max)	Mean ± SD	Median (Min–Max)
Control	66.19 ± 23.06 ^b^	71.07 (38.08–89.41)	92.65 ± 7.05 ^Aa^	89.71 (85.71–103.74)	67.46 ± 19.71 ^ABb^	64.45 (40.97–95.17)
Ethanol	72.95 ± 16.15 ^ab^	67.83 (55.65–97.45)	89.07 ± 15.91 ^Aa^	88.61 (70.39–110.82)	68.99 ± 13.23 ^ABb^	65.01 (54.51–91.09)
Red Wine	61.19 ± 10.51 ^c^	62.18 (47.43–76.81)	106.95 ± 15.45 ^Aa^	106.05 (84.46–130.64)	82.85 ± 14.08 ^Ab^	80.25 (68.20–103.94)
Beer	63.15 ± 15.67	59.54 (48.42–85.37)	54.92 ± 22.34 ^B^	57.43 (18.80–77.43)	59.17 ± 13.91 ^B^	62.40 (39.53–76.62)

Different superscript letters indicate a significant difference between groups stratified by a group (A, B, C, D) and by the experiment duration (a, b, c), for *p* ≤ 0.05.

**Table 13:** The summary of observed influence of the alcoholic beverage and experiment duration on the assessed parameters.

Parameter	Differences Dependent on Alcoholic Beverage		Differences Dependent on Experiment Duration	
	Experiment Duration	Influence of Alcoholic Beverage	Alcoholic Beverage	Influence of Experiment Duration
CAT activity (per mg of protein)	4 weeks	Control ≤ Red wine = Ethanol ≤ Beer	No alcohol (control group)	2 weeks = 4 weeks < 6 weeks
SOD activity (per mg of protein)	2 weeks 4 weeks	Control ≤ Ethanol ≤ Red wine = Beer Beer ≤ Red wine = Control ≤ Ethanol	No alcohol (control group) Ethanol	2 weeks < 4 weeks = 6 weeks 6 weeks ≤ 2 weeks ≤ 4 weeks
GST activity (µM/min per mg of protein)	4 weeks	Beer ≤ Ethanol = Red wine ≤ Control	Red wine	2 weeks < 4 weeks = 6 weeks
Glutathione level (μM per mg protein)	4 weeks 6 weeks	Beer < Ethanol = Control = Red wine Beer ≤ Control = Ethanol ≤ Red wine	No alcohol (control group)	2 weeks = 6 weeks < 4 weeks
			Ethanol	6 weeks ≤ 2 weeks ≤ 4 weeks
			Red wine	2 weeks < 6 weeks < 4 weeks

≤ for assessed parameter, level lower or comparable; < for assessed parameter, level lower; = for assessed parameter, level comparable.
